# Clinical significance of CT angiographic assessment of collateral circulation combined with serum NLRP1 levels in ischemic stroke patients

**DOI:** 10.1097/MD.0000000000033433

**Published:** 2023-03-31

**Authors:** Chong Tao, Yu Wang, Shiyin Xiao

**Affiliations:** a Department of Radiology, Hubei Provincial Hospital of Traditional Chinese Medicine, Wuhan, Hubei, P. R. China; b Department of Radiology, Wuhan Hospital of Traditional Chinese Medicine, Wuhan, Hubei, P. R. China.

**Keywords:** collateral circulation, favorable outcomes, inflammatory factors, ischemic stroke, NLRP1

## Abstract

This research aimed to combine serum NLR-pyrin domain containing 1 (NLRP1) levels and collateral circulation to assess ischemic stroke patients and predict the prognoses of the patients. This present prospective observational study enrolled 196 ischemic stroke patients. All patients underwent CTA as well as digital subtraction angiography (DSA) to assess collateral circulation by American Society of Interventional and Therapeutic Neuroradiology/Society of Interventional Radiology (ASITN/SIR). In addition, we collected serum samples from 100 patients with carotid atherosclerosis as controls. The serum NLRP1, tumor necrosis factor α (TNF-α), interleukin (IL)-6, IL-1β and C-reactive protein (CRP) levels were measured by enzyme-linked immunosorbent assay (ELISA). The age, BMI, sex, smoke condition, diastolic blood pressure, systolic blood pressure, National Institutes of Health Stroke Scores (NIHSS), modified Rankin Scale (mRS) scores, imaging indicators and the levels of triglyceride, total cholesterol (TC), low-density leptin cholesterol (LDLC), high-density leptin cholesterol of all subjects were recorded. All data used SPSS 18.0 to statistical analyses. The serum levels of NLRP1 were remarkably enhanced in the ischemic stroke patients compared with the carotid atherosclerosis patients. The NIHSS score, the mRS score after 90 days and the levels of NLRP1, CRP, TNF-α IL-6 and IL-1β of ischemic stroke patients in the ASITN/SIR grade 0 to 2 group were remarkably elevated than the ischemic stroke patients in ASITN/SIR grade 3 to 4 group. Spearman analysis supported that a positive correlation existed among the NLRP1, CRP, IL-6, TNF-α, and IL-1β levels. The NIHSS score, infarct volume and the levels of NLRP1, IL-6, TNF-α, and IL-1β of ischemic stroke patients in the mRS score ≥ 3 group were remarkably elevated than the ischemic stroke patients in the mRS score ≤ 2 group. ASITN/SIR grade and NLRP1 could be potential diagnostic biomarkers of poor prognosis of ischemic stroke patients. It was found that NLRP1, ASITN/SIR grade, infarct volume, NIHSS, IL-6, and IL-1β were the risk factors for bad prognosis of ischemic stroke patients. This study showed that the serum NLRP1 levels were remarkably decreased in ischemic stroke patients. In addition, the serum NLRP1 levels and ASITN/SIR grade could predict the prognosis of ischemic stroke patients.

## 1. Introduction

Stroke is a major global public health problem, affecting 13.7 million people worldwide.^[1,2]^ According to the World Health Organization, it is the second leading cause of death worldwide, accounting for 11.9% of all deaths.^[3,4]^ The Global Burden of Disease Study 2019 showed that there were 3.94 million new stroke cases, 28.76 million prevalent cases and 2.19 million deaths due to stroke in China in 2019.^[5]^ Stroke is divided into ischemic stroke and hemorrhagic stroke according to pathology and ischemic stroke patients account for about 85% of all stroke patients.^[6]^ Many risk factors are associated with ischemic stroke, including smoking,^[7]^ cardiac causes,^[8]^ hypertension,^[9]^ diabetes mellitus,^[10]^ oxidative stress,^[11]^ inflammation,^[12]^ and so on.^[13]^ A proportion of ischemic stroke patients will have varying degrees of sequelae due to limb dysfunction after treatment, which directly reduces the quality of life of patients and increases the burden on family members and society. Therefore, it is important to evaluate ischemic stroke patients in advance and predict their prognosis.

CT angiography (CTA) and digital subtraction angiography (DSA) are commonly used to assess collateral circulation in patients with ischemic stroke.^[14]^ Previous studies have found that the collateral circulation status of patients with ischemic stroke assessed by imaging usually correlates with the clinical outcomes, and patients with better lateral branch flow tend to have a better prognosis when treated with reperfusion therapy.^[15,16]^ NLR-pyrin domain containing 1 (NLRP1), a protein belonging to the family of the nucleotide-binding domain, leucine-rich repeat (NLR) proteins, was the first to be described as forming an inflammasome.^[17]^ Several studies have reported a pro-inflammatory role for NLRP1 in neurological diseases, cardiovascular diseases, and cancer.^[18–20]^ Inhibition of NLRP1 has been demonstrated to have a protective effect against ischemic stroke in animal studies.^[21,22]^ However, so far, no clinical studies focus on the role of NLRP1 in ischemic stroke development and prognosis.

In the present prospective observational research, we aimed to combine serum NLRP1 levels and collateral circulation to assess ischemic stroke patients and predict the prognosis of the patients. This study might reveal the clinical significance of NLRP1 in ischemic stroke patients, as well as provide novel research targets for ischemic stroke treatment.

## 2. Methods

### 2.1. Subjects

This present prospective observational study enrolled 196 ischemic stroke patients who were admitted from January 2021 to June 2022. All patients were first onset and were diagnosed the ischemic stroke by CT or MRI according to the Chinese guidelines for diagnosis and treatment of acute ischemic stroke 2018.^[23]^ In this guideline, the evaluation and diagnosis of stroke include history and physical examination, National institutes of health stroke scores (NIHSS) assessment of severity, multimodality CT as well as MRI examination of brain lesions versus vascular lesions, and other laboratory tests. The diagnostic criteria in the guideline were: acute onset; focal or global neurological deficit; presence of responsible lesions or symptoms on imaging; exclusion of non-vascular causes; exclusion of cerebral hemorrhage by CT/MRI. In addition, we excluded the following ischemic stroke patients from the study: more than 24 hours from stroke to hospitalization; patients who received anticoagulation therapy within 3 months before the study; patients who were unable to complete the examination and follow-up; patients with serious infection, severe liver, renal, malignancy, ischemic cardiomyopathy, myocardial infarction and unstable angina pectoris; patients with epilepsy, Parkinson disease or other neuropsychiatric disorders. This study did not interfere with patient treatment. All patients were treated with thrombolytic therapy, antiplatelet aggregation, improving circulation, neuroprotection, scavenging free radicals, statins, etc according to the Chinese guidelines for diagnosis and treatment of acute ischemic stroke 2018.^[23]^ In addition, we collected serum samples from 100 patients with carotid atherosclerosis as controls. All carotid atherosclerosis patients were diagnosed by doppler ultrasound (carotid intima-media thickness ≥ 1.0 mm (intimal thickening) or intima-media thickness ≥ 1.5 mm [plaque formation]). An Acuson-Aspen Color Doppler ultrasound diagnostic instrument (GE) with a probe frequency of 7.5 MHz was used to measure the degree of carotid artery stenosis and observed plaques, and the echo condition was regarded as stable (strong echo or medium echo) or unstable (mixed echo or low echo) plaques according to doppler ultrasound. The exclusion criteria of carotid atherosclerosis patients were as follows: patients with silent stroke (infarct foci or softening foci in the brain on initial CT or MRI diagnosis; infarct foci ≥ 3 mm in diameter; no clinical signs or symptoms of neurological deficits associated with the foci; exclusion of other non-vascular diseases and softening foci caused by cerebral hemorrhage); patients with coronary syndrome or other cardiogenic cerebral embolism; patients with cancer, renal or liver dysfunction. Further, 5 ml of fasting cubital venous was collected. All participants signed a written informed consent and were followed up for 3 months. The study was approved by the Ethics Committee of the author hospital No.HHTC20210039.

### 2.2. Imaging examination

CTA was scanned using the GE 64-row CT scanner in a standard protocol. The scan was performed from the aortic arch to the atlantoaxial spine, with 60 to 80 mL of Uvexan (370 mg/mL) and 50 mL of saline pushed into the elbow vein at a flow rate of 5 mL/s. The aortic arch density was monitored using Smart prep technology and the scan was triggered when it reached 150 Hu. The scan parameters were as follows: automatic tube current modulation technique, tube voltage 120 kV, layer thickness 0.625 mm, layer spacing 0.625 mm, Pitch 0.938:1, rotation speed 0.5 s/r.

The DSA images were collected with a Philips DSA imaging system (Philips, Netherlands), and the sequences examined included the frontal and lateral positions of each artery. The internal carotid artery was injected with 8 ml of contrast at a rate of 4 mL/s through the catheter, and the vertebral artery was injected with 6 mL of contrast at a rate of 3 mL/s through the catheter. The exposure is automatically regulated, with a delay of 0.5 seconds. The arterial, cerebral solid and venous phases are acquired at a rate of 6 frames/sec.

Combining 2 imaging modalities to assess collateral circulation by the American Society of Interventional and Therapeutic Neuroradiology/Society of Interventional Radiology (ASITN/SIR).^[24]^ The ASITN/SIR grading is as follows: grading 0, no collateral flow is seen; grading 1, slow collateral flow is seen around the ischemic area, but perfusion defects are still seen in the ischemic area; grading 2, rapid collateral flow is seen around the ischemic area, but partial perfusion defects are still seen in the ischemic area; grading 3, slow collateral flow, complete perfusion of the ischemic area is seen by the end of the venous phase; grading 4, rapid and complete perfusion of the collateral flow into the ischemic area. Good collateral circulation is defined as the ASITN/SIR grading 3 to 4, and poor collateral circulation is the ASITN/SIR grading 0 to 2.

### 2.3. Blood sampling measurement

The serum NLRP1, tumor necrosis factor α (TNF-α), interleukin (IL)-6, IL-1β and C-reactive protein (CRP) levels were measured by enzyme-linked immunosorbent assay (ELISA). Blood samples of fasting cubital venous (5 mL) were collected within 24 hours after admission for all cases. Samples were centrifuged at 2000 g for 15 minutes, followed with ELISA tested using commercially available kits (NLRP1 MBS924094 MyBioSource, IL-6 MBS175877 MyBioSource, CRP MBS177184 MyBioSource, TNF-α MBS824943 MyBioSource, IL-1β MBS175901 MyBioSource). In addition, routing whole blood test was performed using an automatic biochemical analyzer (Hitachi 7600, Hitachi Corporation, Japan) and the levels of interferon-γ and IL-4 were recorded. All these inflammatory factors were measured when the patients were just hospitalized.

### 2.4. Data collection and scale scoring

Demographic and clinical statistics including age, BMI, sex, smoking condition, diastolic blood pressure, systolic blood pressure, etc were collected. Using an automatic biochemical analyzer to a performed whole blood test by Hitachi 7600 of Hitachi Corporation. The total cholesterol (TC), triglyceride, low-density leptin cholesterol (LDLC) and high-density leptin cholesterol levels were recorded. The NIHSS were recorded when the subjects were hospitalized to assess the severity of the stroke. Used the modified Rankin Scale (mRS) to assess the prognosis after 90 days of treatment. mRS score ≥ 3 indicated a bad prognosis and mRS score ≤ 2 indicated a good prognosis.

### 2.5. Statistical analysis

Data were expressed by mean ± SD or median (range) according to distribution, which was confirmed by Kolmogorov-Smirnov analysis. Mann–Whitney test or Student *t* test was used for comparison between the 2 groups. Kruskal–Wallis test or One-way analysis of variance followed by Tukey post hoc test was used for comparison among 3 or more groups. Chi square test was used for rates. Spearman rank correlation was used for correlation analysis. ROC curves were used to assess the diagnostic value of ASITN/SIR grade and NLRP1 for the prognoses of ischemic stroke patients. Logistic regression was performed for risk factors of hypertension. *P* < .05 regarded a significant difference. All data used SPSS 18.0 for statistical analyses.

## 3. Results

### 3.1. Clinical characteristics of all participants

**Figure 1.**
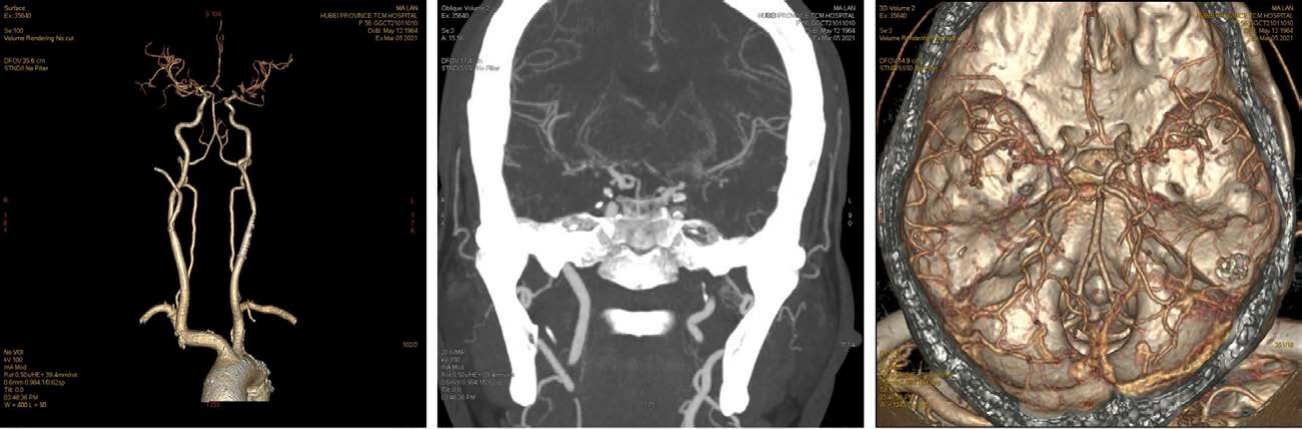
Multiple moderate to severe stenosis of the anterior and middle cerebral arteries—54 years old—female.

This study enrolled 196 ischemic stroke patients and 100 carotid atherosclerosis patients. There were 59 carotid atherosclerosis patients with arterial stenosis < 70%, and 41 carotid atherosclerosis patients with arterial stenosis ≥ 70%. 55 stable plaques and 45 unstable plaques in patients with carotid atherosclerosis. All ischemic stroke patients underwent imaging evaluation (Fig. 1). In addition, all subjects were evaluated collateral circulation by ASITN/SIR and divided into 2 groups including ASITN/SIR grade 0 to 2 (n = 86) group and ASITN/SIR grade 3 to 4 (n = 110) group. Compared the demographic and clinical data of the 2 groups when the patients hospitalized, we found that no significant differences in age, sex, BMI, smoking proportion and levels of TC, triglyceride, high-density leptin cholesterol and LDLC between 2 groups (Table 1). The NIHSS score, the mRS score after 90 days and the infarct volume of ischemic stroke patients in the ASITN/SIR grade 0 to 2 group were remarkably elevated than the ischemic stroke patients in ASITN/SIR grade 3-4 group (*P* < .05).

**Table 1:** Basic characteristics of all patients.

Variable	ASITN/SIR grade 3–4 group, n = 110	ASITN/SIR grade 0–2 group, n = 86	*P* value
Age, yr	58 (44–74)	59 (41–78)	.532
Sex, female (%)	52 (47.27)	39 (45.35)	.778
BMI	25.83 (20.88–29.30)	25.44 (20.89–29.16)	.134
Current smoker, n (%)	47 (42.73)	39 (45.39)	.887
Hypertension, n (%)	71 (65.55)	64 (74.42)	.280
Hyperlipidemia, n (%)	19 (17.27)	20 (23.26)	.377
Diabetes, n (%)	28 (25.45)	25 (29.07)	.633
NIHSS	5.00 (1–20)	11 (1–20)	<.001
TC (mmol/L)	4.52 (2.85–5.59)	4.36 (2.94–5.57)	.746
TG (mmol/L)	1.26 (0.78–1.62)	1.24 (0.79–1.63)	.642
HDLC (mmol/L)	1.10 (0.73–1.35)	1.03 (0.72–1.31)	.134
LDLC (mmol/L)	2.85 ± 0.48	2.82 ± 0.52	.643
Infarct volume (cm^3^)	0.83 (0.19–4.71)	1.75 (0.08–5.07)	<.001
mRS after 90 d	1.00 (0–5)	3.00 (0–5)	<.001

ASITN/SIR = American Society of Interventional and Therapeutic Neuroradiology/Society of Interventional Radiology, BMI = body mass index, HDLC = high-density leptin cholesterol, LDLC = low-density leptin cholesterol, mRS = modified Rankin Scale, NIHSS = National Institutes of Health Stroke Scores, TC = total cholesterol, TG = triglyceride.

### 3.2. Comparisons of NLRP1 and inflammatory factors in ischemic stroke patients

**Figure 2.**
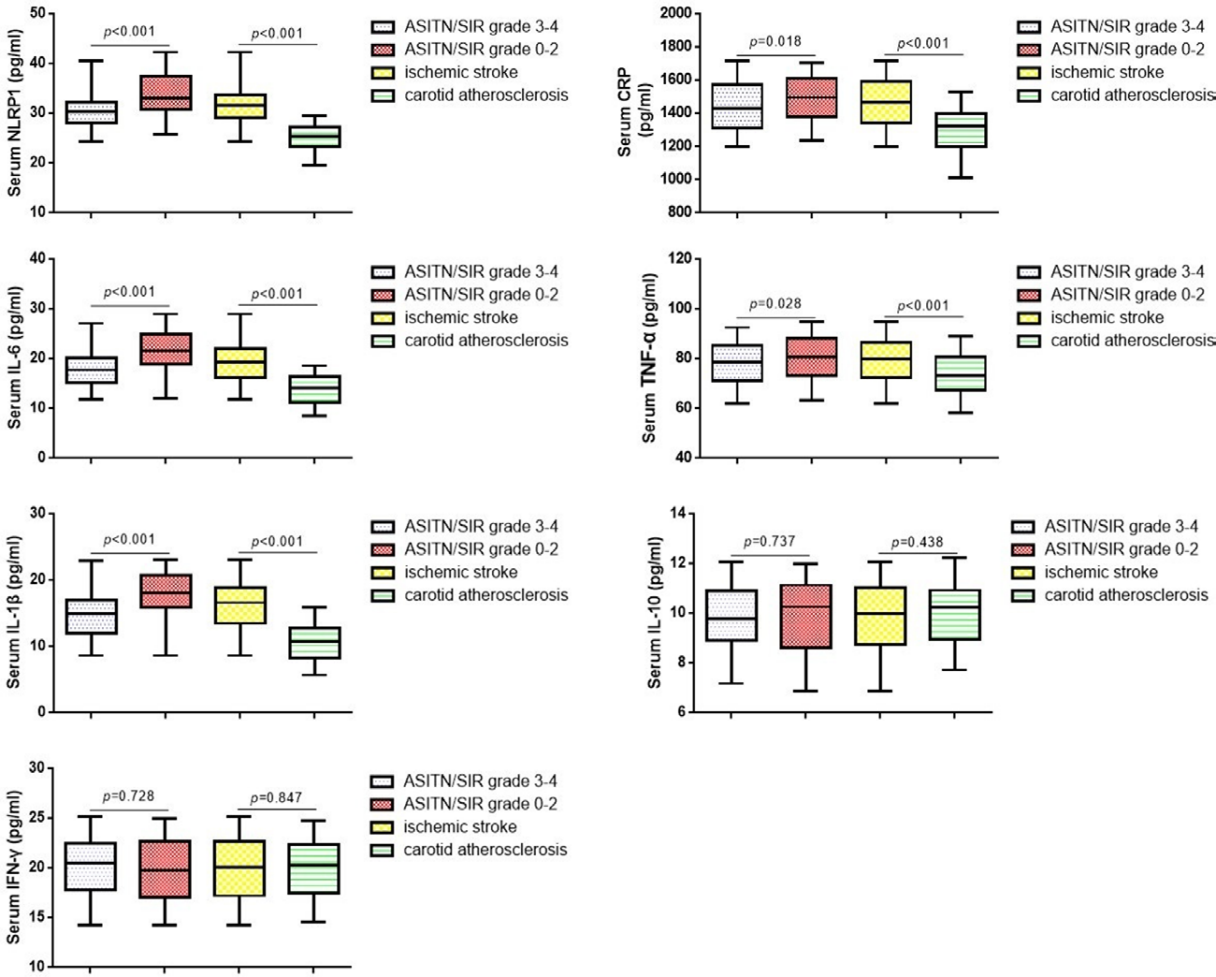
Comparisons of NLRP1 and inflammatory factors in all subjects. NLRP1 = NLR-pyrin domain containing 1.

To further investigate the relationship between NLRP1 and inflammatory factors in ischemic stroke patients, we measured the serum levels of NLRP1, CRP, IL-6, TNF-α, and IL-1β by ELISA. As shown in Figure 2, the serum levels of NLRP1, CRP, IL-6, TNF-α, and IL-1β levels were remarkably enhanced in the ischemic stroke patients compared with the carotid atherosclerosis patients, while no significant differences in serum IL-10 and interferon-γ levels were found between 2 groups. In addition, the serum levels of NLRP1, CRP, IL-6, TNF-α, and IL-1β in the ASITN/SIR grade 0 to 2 group were significantly increased compared with the ASITN/SIR grade 3 to 4 group. No significant differences in serum CRP and TNF-α levels were found between the 2 groups. Spearman analysis supported that a positive correlation existed among the NLRP1, CRP, IL-6, TNF-α, and IL-1β levels (Table 2).

**Table 2:** Correlation analysis among NLRPI and inflammatory factors.

	NLRP1	CRP	IL-6	TNF-α	IL-1β	IL-10	IFN-γ
NLRP1							
Spearman correlation	1.000	0.171	0.547	0.173	0.468	−0.084	0.020
*P*		.016	<.001	.016	<.001	.242	.780
CRP							
Spearman correlation	0.171	1.000	0.047	−0.049	0.106	−0.008	−0.070
*P*	.016		.510	.491	.139	.913	.327
IL-6							
Spearman correlation	0.547	0.047	1.000	0.229	0.519	−00.09	−0.081
*P*	<.001	.510		.001	<.001	.896	.260
TNF-α							
Spearman correlation	0.173	−0.049	0.229	1.000	0.063	0.127	−0.078
*P*	.016	.491	.001		.383	.077	.275
IL-1β							
Spearman correlation	0.468	0.106	0.519	0.063	1.000	−0.087	0.009
*P*	<.001	.139	<.001	.383		.226	.897
IL-10							
Spearman correlation	−0.084	−0.008	−0.009	0.127	−0.087	1.000	−0.047
*P*	.242	.913	.896	.077	.226		.511
IFN-γ							
Spearman correlation	0.020	−0.070	−0.081	−0.780	0.009	−0.047	1.000
*P*	.780	.327	.260	.275	.897	.511	

CRP = C-reactive protein, IFN-γ = interferon-γ, IL = interleukin, NLRP1 = NLR-pyrin domain containing 1, TNF-α = tumor necrosis factor α.

### 3.3. Connection of serum NLRP1 levels, clinical data, and prognosis of ischemic stroke patients

All ischemic stroke patients measured mRS scores after 90 days of treatment and were divided into 2 groups including mRS score ≤ 2 group and mRS score ≥ 3 group. Compared the demographic and clinical data of the 2 groups when the patients were hospitalized, we found no significant differences in age, sex, BMI, smoking proportion and levels of TC and LDLC between the mRS score ≤ 2 group and mRS score ≥ 3 group (Table 3). The NIHSS score, infarct volume and the levels of NLRP1, IL-6, TNF-α, and IL-1β of ischemic stroke patients in the mRS score ≥ 3 group were remarkably elevated than the ischemic stroke patients in the mRS score ≤ 2 group (*P* < .05). However, the ASITN/SIR grades in the mRS score ≥ 3 group were significantly lower compared with the mRS score ≤ 2 group (*P* < .05).

**Table 3:** Serum NLRP1 levels and clinical data in ischemic stroke patients with different prognosis.

Variable	mRS score ≤ 2 group, n = 128	mRS score ≥ 3 group, n = 68	*P* value
Age, yr	58 (41–74)	60 (44–78)	.440
Sex, female (%)	61 (47.66)	30 (44.12)	.670
BMI	25.46 ± 2.26	25.13 ± 2.33	.328
Current smoker, n (%)	55 (42.97)	31 (45.59)	.776
NIHSS	5 (1–17)	14 (4–20)	<.001
Hypertension, n (%)	85 (66.41)	50 (73.53)	.280
Hyperlipidemia, n (%)	22 (17.19)	17 (25.00)	.224
Diabetes, n (%)	32 (25.00)	21 (30.88)	.431
Infarct volume (cm^3^)	0.80 ± 0.29	2.61 ± 1.28	<.001
TC (mmol/L)	4.51 (2.85–5.590	4.37 (2.94–5.57)	.598
TG (mmol/L)	1.24 (0.78–1.56)	1.27 (0.82–1.63)	.052
LDLC (mmol/L)	2.84 ± 0.19	2.81 ± 0.51	.684
HDLC (mmol/L)	1.08 (0.73–1.35)	1.05 (0.72–1.33)	.453
CRP (pg/mL)	1438.29 (1198.9–1714.47)	1486.64 (1234.85–1702.37)	.131
IL-6 (pg/mL)	17.25 (11.85–22.18)	23.53 (16.75–28.98)	<.001
TNF-α (pg/mL)	78.57 (61.85–92.58)	81.91 (66.43–94.73)	.011
IL-1β (pg/mL)	14.17 (8.63–19.20)	20.19 (15.14–23.05)	<.001
NLRP1 (pg/ml)	30.03 (24.32–33.92)	35.63 (28.97–42.33)	<.001
IL-10 (pg/ml)	9.78 (7.18–12.07)	9.85 (6.86–11.97)	.584
IFN-γ (pg/ml)	20.53 (14.23–25.19)	19.80 (14.24–24.96)	.507
ASITN/SIR grade	3 (1–4)	2 (1–4)	<.001

ASITN/SIR = American Society of Interventional and Therapeutic Neuroradiology/Society of Interventional Radiology, BMI = body mass index, CRP = C-reactive protein, HDLC = High-density leptin cholesterol, IFN-γ = interferon-γ, IL = interleukin, LDLC = low-density leptin cholesterol, **mRS** = modified Rankin Scale, NIHSS = National Institutes of Health Stroke Scores, NLRP1 = NLR-pyrin domain containing 1, TC = total cholesterol, TG = triglyceride, TNF-α = tumor necrosis factor α.

### 3.4. Diagnostic value of ASITN/SIR grade and NLRP1 for poor prognosis of ischemic stroke patients

**Figure 3.**
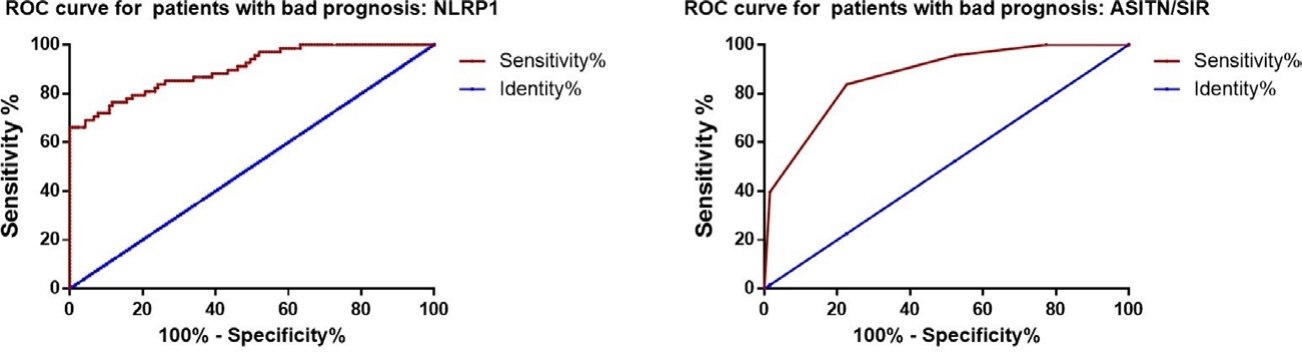
ROC curves for NLRP1 and ASITN/SIR grade in diagnostic poor prognosis of ischemic stroke. ASITN/SIR = American Society of Interventional and Therapeutic Neuroradiology/Society of Interventional Radiology, NLRP1 = NLR-pyrin domain containing 1, ROC = receiver operating characteristic.

We draw ROC curves to assess the diagnostic value of ASITN/SIR grade and NLRP1 for the prognoses of ischemic stroke patients. The result showed that ASITN/SIR grade and NLRP1 could be potential diagnostic biomarkers of poor prognosis of ischemic stroke patients (Fig. 3), the AUC of NLRP1 was 0.902, cutoff value 32.11 pg/mL, sensitivity 80.1%, specificity 78.9% and the AUC of ASITN/SIR grade was 0.871, cutoff value 2.5, sensitivity 83.8%, specificity 77.3%.

### 3.5. Risk factors of poor prognosis of ischemic stroke patients by logistic regression analysis

The risk variables for poor prognosis in ischemic stroke patients were calculated using binary regression analysis. It was found that NLRP1, ASITN/SIR grade, infarct volume, NIHSS, IL-6, and IL-1β were the risk factors for bad prognosis of ischemic stroke patients (Table 4).

**Table 4:** Risk factors of ischemic stroke patients with bad prognosis by logistic regression analysis.

Variables	Wald	Odds ratio	95% CI	*P* value
Age	0.087	0.992	0.941–1.046	.768
BMI	0.418	0.930	0.745–1.160	.518
CRP	1.347	0.992	0.980–1.005	.246
IL-6	5.128	2.452	1.128–5.327	.024
TNF-α	1.011	1.128	0.892–1.425	.315
IL-1β	3.944	12.367	1.033–148.011	.047
TC	0.009	0.966	0.481–1.940	.923
TG	2.215	5.812	0.573–58.988	.137
HDLC	0.161	1.784	0.106–30.173	.688
LDLC	1.136	1.850	0.597–5.736	.286
Infarct volume	34.495	26.035	8.733–77.257	<.001
NLRP1	4.702	2.899	1.107–7.537	.030
NIHSS	28.839	1.664	1.382–2.004	<.001
ASITN/SIR grade	15.286	2.934	1.710–5.032	<.001

ASITN/SIR = American Society of Interventional and Therapeutic Neuroradiology/Society of Interventional Radiology, BMI = body mass index, CRP = C-reactive protein, HDLC = High-density leptin cholesterol, IL = interleukin, LDLC = low-density leptin cholesterol, NIHSS = National Institutes of Health Stroke Scores, NLRP1 = NLR-pyrin domain containing 1, TC = total cholesterol, TG = triglyceride, TNF-α = tumor necrosis factor α.

## 4. Discussion

Although intravenous thrombolysis and thrombectomy can be effective in the treatment of ischemic stroke, there are still many patients with poor prognoses during treatment and recovery. It has been reported that stroke is the leading cause of disability in adults and about 90% of stroke patients are left with some residual deficit.^[25]^ Thus, it is urgent to develop new biomarkers and comprehensive approaches to screen ischemic stroke patients with worse prognoses in advance. In this research, we found that the serum levels of NLRP1 and collateral circulation could predict the prognosis of ischemic patients. In recent years, an increasing number of studies have focused on the effect of collateral circulation in patients with ischemic stroke. Malhotra et al confirmed that collateral circulation status and infarct volume are independent predictors of functional outcomes in patients with ischemic stroke.^[26]^ A clinical study by Peng et al suggested that multi-phase CT angiography cerebral collateral circulation score independently predicted neurological prognosis in ischemic stroke patients.^[27]^ A retrospective study of 686 patients with ischemic stroke by Conrad et al supported that whole-brain collateral circulation status and the presence of atherosclerosis were the most relevant predictors of clinical and radiological outcomes.^[28]^ Chang et al found that combined blood pressure variability parameters and collateral circulation grading are important predictors of functional prognosis at 3 months in patients with ischemic stroke.^[15]^ ASITN/SIR collateral score is the recommended and most widely used angiographic score in endovascular acute stroke trials. High collateral grades defined using the ASITN/SIR scale are associated with improved reperfusion rates after endovascular interventions.^[29,30]^ The ASITN/SIR scoring systems have shown a good correlation with clinical outcomes.^[31]^ Similar results were obtained in our research, in which we found that grading of collateral circulation assessment was a risk factor for poor prognosis in patients with ischemic stroke and could be used to predict poor patient outcomes.

Cerebral ischemia promotes the production of pro-inflammatory mediators and induces cell death and cellular dysfunction, thereby inducing neuroinflammation. Excessive inflammation can produce neurotoxins and cerebral edema. Post-stroke immune response has recently emerged as a breakthrough target for ischemic stroke therapeutic strategies.^[32,33]^ Previous studies have found that the inflammatory factors IL-6 and IL-1β are strongly associated with the prognosis of ischemic stroke patients.^[34,35]^ In addition, NLRP1 plays a role in various diseases by producing the inflammatory factors IL-6 and IL-1β.^[36]^ More recent studies have focused on the inflammation-inducing role of NLRP1 in various diseases and its association with patient prognosis. Marina et al suggested that NLRP1 activation causes neuroinflammation that is associated with Alzheimer disease, and inhibiting NLRP1 activation may be used to treat Alzheimer disease.^[37]^ Cao et al determined that miR-9a-5p is involved in NLRP1-mediated ischemic injury and overexpression of miR-9a-5p can regulate NLRP1 to ameliorate brain injury after ischemic stroke in an ischemic stroke rat model.^[38]^ Ma et al indicated that isoproterenol exerts neuroprotective effects in stroke through inhibition of NLRP1-induced inflammatory pathways.^[39]^ Wang et al showed that the levels of NLRP3 and inflammatory factors were significantly higher than health controls.^[40]^ These results were similar to our results, in which we did not have a healthy control group but included patients with carotid atherosclerosis as controls, showing the serum levels of CRP, IL-6, TNF-α, and IL-1β were remarkably enhanced in the ischemic stroke patients compared with the carotid atherosclerosis patients. However, there are no more clinical studies on the role of NLRP1 in patients with ischemic stroke. In our study, we found that serum NLRP1 levels was decreased in ischemic stroke patients with poor prognosis and was associated with other inflammatory factors as well as collateral circulation.

This present research also has some limitations. First, we only included a small size of the study population. Secondly, we only checked a small number of inflammatory factors. Thirdly, the molecular mechanism of NLRP1 affecting ischemic stroke development is unclear. Finally, the treatment of all ischemic stroke patients was not completely consistent.

## 5. Conclusion

This study showed that the serum NLRP1 levels were remarkably decreased in ischemic stroke patients. In addition, the serum NLRP1 levels and ASITN/SIR grade could predict the prognosis of ischemic stroke patients. This study may provide a new approach to screening ischemic stroke patients with worse prognoses in advance.

## Author contributions

**Formal analysis:** Chong Tao.

**Writing – original draft:** Shiyin Xiao.

**Writing – review & editing:** Yu Wang.
